# Pharmacodynamics of sustained levels of PTH following palopegteriparatide treatment in adults with hypoparathyroidism

**DOI:** 10.1210/jendso/bvag098

**Published:** 2026-04-18

**Authors:** Aliya A Khan, Lars Rejnmark, Rajesh Jain, Susanne Pihl, Vibeke Miller Breinholt, Carol Zhao, Bryant Lai, Jenny Ukena, Christopher T Sibley, Aimee D Shu

**Affiliations:** Division of Endocrinology and Geriatrics, McMaster University, Hamilton, ON, Canada L8S 4L8; Department of Clinical Medicine, Department of Endocrinology and Internal Medicine, Aarhus University Hospital, 8200 Aarhus N, Denmark; Division of Biological Sciences, University of Chicago, Chicago, IL 60637, USA; Ascendis Pharma A/S, 2900 Helllerup, Denmark; Ascendis Pharma A/S, 2900 Helllerup, Denmark; Ascendis Pharma, LLC, Palo Alto, CA 94304, USA; Ascendis Pharma, LLC, Palo Alto, CA 94304, USA; Ascendis Pharma, LLC, Palo Alto, CA 94304, USA; Ascendis Pharma, LLC, Palo Alto, CA 94304, USA; Ascendis Pharma, LLC, Palo Alto, CA 94304, USA

**Keywords:** PTH(1-34), transCon PTH, hypoparathyroidism, palopegteriparatide, parathyroid hormone, replacement therapy

## Abstract

**Purpose:**

Palopegteriparatide is a prodrug of parathyroid hormone (PTH) (1-34), administered once daily. It is designed to provide active PTH within the physiologic range for 24 hours/day. The effects of palopegteriparatide on serum and urine markers were explored to determine if the pharmacodynamic profile of palopegteriparatide reflects continuous disease control in adults with hypoparathyroidism.

**Methods:**

A 24-hour sub-study was conducted during the PaTH Forward trial. Eligible participants were those who completed the Week 58 visit and received stable doses of palopegteriparatide for the prior week. Upon admission to the clinical research unit, participants received their usual dose of palopegteriparatide. Plasma PTH, serum calcium (albumin-adjusted), and serum phosphate concentrations were evaluated at 4-hour intervals. Urinary calcium, phosphate, and citrate concentrations were determined from each of six 4-hour collection periods. Descriptive statistics were performed.

**Results:**

Palopegteriparatide doses ranged from 12 to 33 µg/day and resulted in continuous exposure to active PTH within the estimated normal range over 24 hours. Mean serum calcium and serum phosphate were maintained near or within the normal ranges. Median urine calcium concentration was stable in the normal range for all 4-hour collection periods. Urine phosphate concentrations showed minor variability across collection intervals, and urine citrate was maintained above the lower limit of normal.

**Conclusion:**

Palopegteriparatide provided continuous exposure to active PTH within the physiological range. The expected pharmacodynamic effects of continuous PTH exposure on serum and urine chemistries were consistent with the efficacy and safety of palopegteriparatide demonstrated in the published PaTH Forward and PaTHway clinical trials.

Parathyroid hormone (PTH) is the primary regulator of calcium-phosphate balance [1]. Patients with hypoparathyroidism, a rare endocrine disease characterized by absent or insufficient production of PTH, exhibit low serum calcium, high serum phosphate, elevated fractional excretion of calcium in the urine, and reduced bone turnover [2]. Conventional therapy for hypoparathyroidism, active vitamin D, and elemental calcium may alleviate hypocalcemia but does not restore PTH-dependent renal tubular calcium reabsorption and phosphate excretion or PTH-dependent skeletal metabolism [3]. Management with active vitamin D and calcium, moreover, elevates the risk for hypercalciuria and may increase the likelihood of associated complications such as nephrolithiasis or nephrocalcinosis relative to patients without hypoparathyroidism [4-6]. Patients receiving conventional therapy also report significant pill burden and negative impacts on health-related quality of life [7-9].

Replacement therapy with exogenous PTH offers the potential to address the root cause of hypoparathyroidism. However, the short half-life of endogenous PTH (estimated at 2 to 4 minutes) presents a challenge in its delivery as a therapeutic option [10, 11]. Once-daily dosing of short-lived PTH therapies may result in wide fluctuations of PTH exposure and serum calcium levels within a 24-hour time period, along with inadequate control of urinary calcium excretion [12-16]. The ideal therapy for hypoparathyroidism would be a PTH replacement that mimics tonic endogenous exposure consistently within the physiologic range, thereby enabling consistent maintenance of calcium homeostasis [12, 14].

Palopegteriparatide is a prodrug of PTH(1-34) administered once daily and designed to provide active PTH within the physiological range for 24 hours/day. Palopegteriparatide consists of PTH(1-34), bound to an inert methoxypolyethylene glycol carrier by a cleavable linker [13, 17]. Upon exposure to physiological pH and temperature, autocleavage of the linker occurs, and active PTH is released in a sustained manner, while the inert carrier and its covalently attached linker are excreted by the kidneys [13, 14, 17]. In a phase 1 trial in healthy adults administered palopegteriparatide, the apparent half-life of released PTH was determined to be approximately 60 hours [17]. Based on the results of the phase 2 PaTH Forward and phase 3 PaTHway trials [18, 19], palopegteriparatide has been approved in the United States, Europe, and other regions for the treatment of hypoparathyroidism in adults.

To further elucidate the pharmacokinetic profile of palopegteriparatide and its pharmacodynamic effects on serum and urine chemistries over 24 hours in the target population, we conducted a sub-study of participants in the phase 2 PaTH Forward trial. The results of this sub-study are reported here.

## Methods

A complete description of the PaTH Forward trial material, methods, and results has been previously published [20]. Briefly, PaTH Forward was a phase 2, randomized, double-blind placebo-controlled trial through Week 4, followed by an open-label extension period through Week 266 that evaluated the safety, tolerability, and efficacy of once-daily palopegteriparatide in adults with hypoparathyroidism. The protocol was reviewed by appropriate institutional review boards and ethics committees, and all participants provided signed informed consent prior to initiation (ClinicalTrials.gov registration number NCT04009291; EudraCT number 2018-004815-33). A sub-study of PaTH Forward participants was conducted to further characterize the pharmacodynamic profile of palopegteriparatide in adults with hypoparathyroidism.

Eligible participants for the sub-study were enrolled in the PaTH Forward open-label extension, had passed the Week 58 visit, had taken a stable dose of palopegteriparatide for the last ≥7 days, and had agreed to a 24-hour admission to a clinical research unit. Palopegteriparatide dosing was individualized based on serum calcium levels, using a protocol-specified algorithm. Of the 12 eligible participants, 7 were administered their usual dose of palopegteriparatide upon admission to the clinical research unit (timepoint 0). Five of the 12 participants received their usual dose of palopegteriparatide at their usual dosing time, which was not aligned with the time of admission. Therefore, complete 24-hour post-dose sampling was not available for these 5 participants. Data from the 7 participants who had completed 24-hour post-dose sampling are reported in the Results section.

The primary analyte of interest was active PTH in plasma (ie, sum of free PTH(1-34) and its metabolite, free PTH(1-33)), which represents the total amount of PTH released from the prodrug. The estimated normal range for PTH(1-34) is 4 to 26 pg/mL, calculated from the normal range for PTH(1-84) of 10 to 65 pg/mL and the 60% lower molecular weight of PTH(1-34) [17, 21]. Although it was not an eligibility requirement, all participants in this sub-study had achieved independence from active vitamin D after more than a year of palopegteriparatide treatment. No participants were taking active vitamin D or inactive vitamin D3 during the sub-study period. Intake of oral calcium supplements was recorded throughout the study, along with concurrent medications that had the potential to influence calcium metabolism.

Blood sampling for active PTH began immediately prior to the palopegteriparatide dose and was performed every 4 hours thereafter. Active PTH was quantified in acidified human ethylenediaminetetraacetic acid (EDTA) plasma via a validated assay. Acidification of the blood with an acidic citrate buffer solution (pH 4) immediately upon sampling was required to minimize the release of PTH from the prodrug. The acidified EDTA plasma samples were processed by protein precipitation followed by liquid–liquid extraction, and subsequent solid-phase extraction. PTH(1-34), PTH(1-33), and the stable isotope-labeled internal standards were analyzed by ultra-high-performance liquid chromatography with tandem mass spectrometry (UHPLC–MS/MS). Validated assay ranges for Free PTH(1-34) and Free PTH(1-33) were 1.00 to 200 pg/mL and 0.684 to 137 pg/mL, respectively (data on file). Mass transitions for both peptides were collected simultaneously, and active PTH was only calculated when both PTH(1-34) and PTH(1-33) were above the lower limit of quantification. The pharmacokinetic evaluation for released PTH was conducted using neat plasma samples. There was no interference from the prodrug (palopegteriparatide) in the assay.

Serum and urine chemistries were evaluated using standard laboratory methods. Descriptive statistics were performed. Serum calcium (albumin-adjusted) and phosphate concentrations were measured from samples drawn at 4-hour intervals and reported as mean values along with standard error (SE). Urinary calcium, phosphate, and citrate concentrations were determined from each of six 4-hour collection periods and reported as median (interquartile range [IQR]) due to skewness of the data.

## Results

Twelve participants were enrolled in this sub-study. Seven of the 12 participants administered their usual dose of palopegteriparatide upon admission to the clinical research unit (timepoint 0), per protocol. Five of the 12 participants received their usual dose of palopegteriparatide at their usual dosing time, which was not aligned with the time of admission. Therefore, complete 24-hour post-dose sampling was not available for these 5 participants. Data from the 7 participants who had completed 24-hour post-dose sampling are reported below.

**Figure 1:**
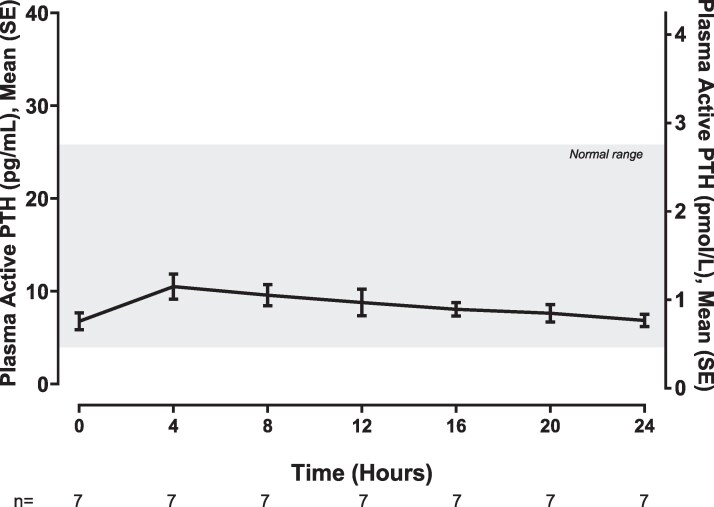
24-hour plasma profile of active PTH, as released from palopegteriparatide. Timepoint 0 represents the time of dosing for the PaTH Forward sub-study. The estimated normal range for PTH(1-34), 4 to 26 pg/mL, was calculated based on the normal range for PTH(1-84) of 10 to 65 pg/mL and a 60% lower molecular weight of PTH(1-34). Abbreviations: PTH, parathyroid hormone; SE, standard error.

**Figure 2:**
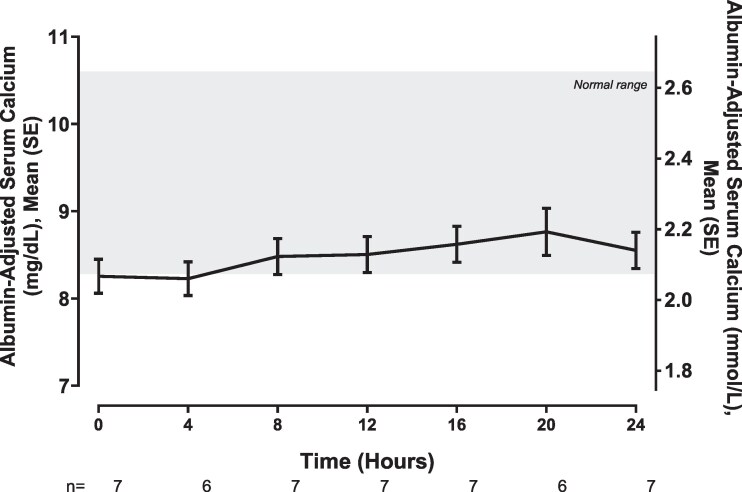
24-hour profile of albumin-adjusted serum calcium in palopegteriparatide-treated participants. Timepoint 0 represents the time of dosing for the PaTH Forward sub-study. The normal range for albumin-adjusted serum calcium was 8.3 to 10.6 mg/dL. Abbreviation: SE, standard error.

**Figure 3:**
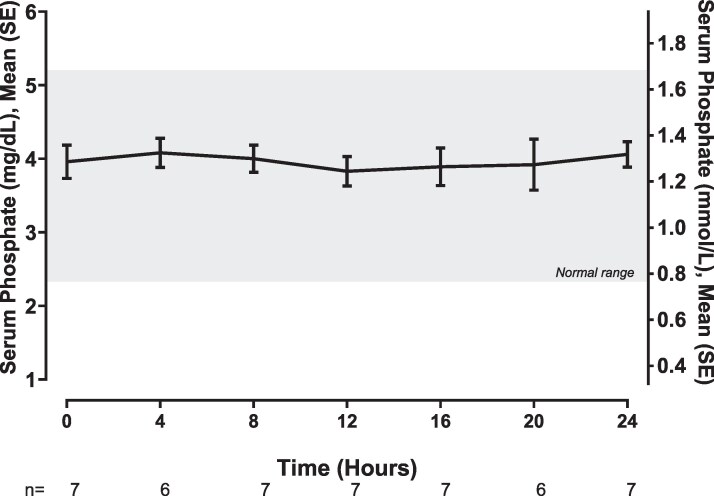
24-hour profile of serum phosphate in palopegteriparatide-treated participants. Timepoint 0 represents the time of dosing for the PaTH Forward sub-study. The normal range for serum phosphate was 2.2 to 5.1 mg/dL. Abbreviation: SE, standard error.

Demographic and baseline clinical characteristics of sub-study participants were consistent with the overall PaTH Forward population (Table 1). Doses of palopegteriparatide for sub-study participants by Week 58 of PaTH Forward ranged from 12 to 33 µg/day and resulted in continuous exposure to active PTH within the calculated estimated normal range (4-26 pg/mL) over 24 hours (Fig. 1). Mean (SE) active PTH concentration was 6.8 (0.9) pg/mL at dosing, reached a peak of 10.5 (1.4) pg/mL at 4 hours, and decreased gradually over the next 20 hours to 6.9 (0.7) pg/mL. The mean peak-to-trough ratio of 1.5 reflected sustained release of PTH(1-34) within the physiologic range with the once-daily dosing regimen. An elaborate PK assessment was not performed because samples were collected under steady-state conditions with minimal peak-to-trough fluctuations. Therefore, only the above PK-parameters are deemed relevant to convey. Mean serum calcium was maintained near or within the normal range over the 24-hour study period (Fig. 2). Mean (SE) serum calcium concentration was 8.3 (0.2) mg/dL at dosing and was maintained between 8.2 (0.2) and 8.8 (0.3) mg/dL over 24 hours. Mean serum phosphate also remained stable within the normal range for 24 hours (Fig. 3). Mean (SE) serum phosphate concentration was 4.0 (0.2) mg/dL at dosing and was maintained between 3.8 (0.2) and 4.1 (0.2) mg/dL for 24 hours.

**Table 1:** Demographics and baseline clinical Characteristics

	Sub-study cohort (*n* = 7)	All PaTH Forward participants (*N* = 59) [20]
**Mean age, years (SE)**	50 (4)	50 (2)
**Age group, *n* (%)**		
<30 years	0	3 (5)
≥30 to <65 years	6 (86)	51 (86)
≥65 years	1 (14)	5 (9)
**Sex, female, *n* (%)**	6 (86)	48 (81)
**Race, white, *n* (%)**	6 (86)	54 (92)
**Geographic region, *n* (%)**		
North America	7 (100)	38 (64)
Europe	0	21 (36)
**Etiology, *n* (%)**		
Acquired from neck surgery	6 (86)	47 (80)
Autoimmune disease	0	1 (2)
Idiopathic disease	1 (14)	11 (19)
**Mean duration of hypoparathyroidism, years, (range)**	11 (3-30)	12 (1-39)

Abbreviation: SE, standard error.

**Figure 4:**
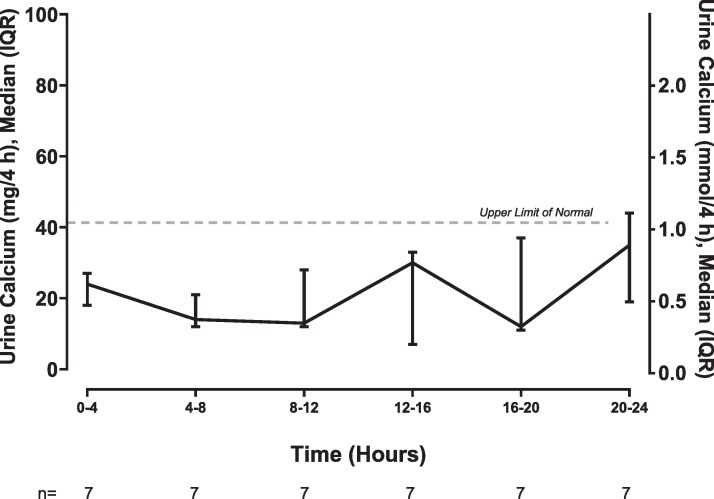
24-hour profile of urine calcium excretion in palopegteriparatide-treated participants. Timepoint 0 represents the time of dosing for the PaTH Forward sub-study. The upper limit of normal for each collection was defined as 42 mg/4 hours and was derived from a daily upper limit of normal (250 mg/24 hours) divided into 6 consecutive intervals of 4 hours each. Abbreviation: IQR, interquartile range.

**Figure 5:**
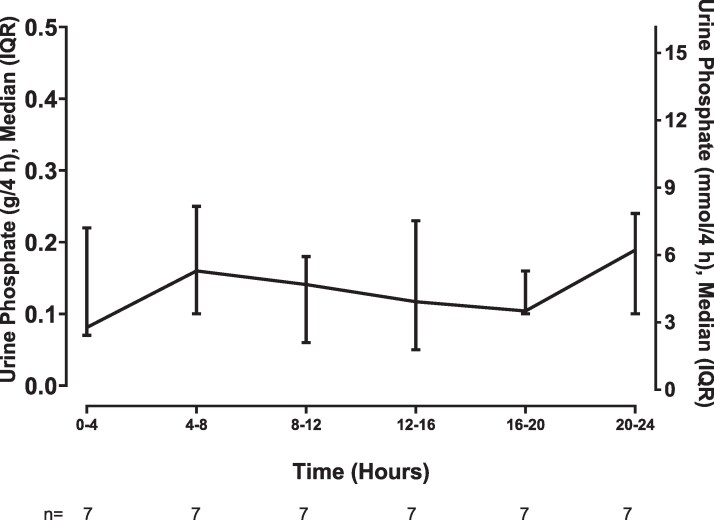
24-hour profile of urine phosphate excretion in palopegteriparatide-treated participants. Timepoint 0 represents the time of dosing for the PaTH Forward sub-study. Abbreviation: IQR, interquartile range.

**Figure 6:**
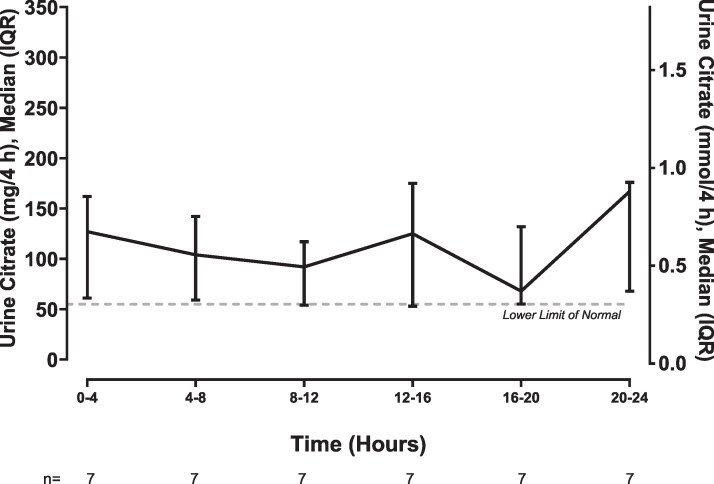
24-hour profile of urine citrate excretion in palopegteriparatide-treated participants. Timepoint 0 represents the time of dosing for the PaTH Forward sub-study. Hypocitraturia was defined as urinary citrate excretion <320 mg per day. The lower limit of normal for each collection was defined as 53 mg/4 hours and was derived from the <320 mg daily threshold divided into 6 intervals. Abbreviation: IQR, interquartile range.

Median urine calcium concentration was stable in the normal range for all 4-hour collection intervals (Fig. 4), reflecting the control conferred by long-term treatment with palopegteriparatide. Median (IQR) urine calcium ranged from 12 (11, 37) to 35 (19, 44) mg per 4-hour interval. Median urine phosphate showed some variability across the collection intervals and may have been influenced by external factors such as participant dietary intake, hydration, and activity (Fig. 5). Median (IQR) urine phosphate ranged from 0.08 (0.07, 0.22) to 0.19 (0.10, 0.24) g per 4-hour interval. Median (IQR) urine citrate also demonstrated minor variability across collection periods, ranging from 68 (55, 132) to 167 (68, 176) mg per 4-hour interval (Fig. 6). Throughout the 24-hour assessment period, median values for urine calcium, phosphate, and citrate were all within normal ranges.

## Discussion

Recently published best practice recommendations for the diagnosis and management of hypoparathyroidism highlight the concept that physiologic PTH replacement may result in improved clinical outcomes compared to the use of conventional therapy for hypoparathyroidism [22]. While management with active vitamin D and high doses of calcium may increase serum calcium, conventional therapy is associated with fluctuating calcium levels and may increase urinary calcium and associated risks of nephrolithiasis, nephrocalcinosis, and renal impairment [4, 23-25]. Patients managed with conventional therapy can have persistent reductions in quality of life and experience impaired physical functioning and well-being, despite normal serum calcium levels [26-28], suggesting that PTH may be important for neurocognitive function [29]; quality of life may be influenced through effects of insufficient PTH on the central nervous system [22, 30].

Short-lived PTH therapies have limitations with respect to ensuring stability of serum PTH and calcium levels. With a median plasma half-life of 2.2 hours, subcutaneous recombinant human PTH (rhPTH[1-84]) 100 µg administered once daily as an adjunct to conventional therapy has been associated with a supraphysiologic peak concentration of PTH, followed by a decline and a return to low PTH levels within 12 hours of dosing [15]. In addition, significant serum calcium fluctuations over 24 hours have been reported with this treatment approach, including hypercalcemic periods of 6 to 12 hours post injection [15]. With a half-life of 1 hour, teriparatide delivered via subcutaneous injection reaches peak serum concentrations within 30 minutes with a decline in concentration to non-quantifiable levels within 3 hours of injection [31].

In this steady-state evaluation of palopegteriparatide and its effects in adults with hypoparathyroidism, active PTH levels were maintained within the lower half of the physiological range following continued palopegteriparatide administration. The findings of this sub-study were consistent with those of a phase 1 study in healthy adults receiving palopegteriparatide that demonstrated a physiologic steady-state pharmacokinetic profile of active PTH [17]. Rich sampling generated a depth of information from serum and urine laboratory values, demonstrating that palopegteriparatide stabilized calcium and phosphate throughout the 24-hour dosing period. These findings reflect the systemic effects PTH replacement therapy. The long apparent half-life of PTH released from palopegteriparatide supports stable serum calcium levels over 24 hours when administered once daily. Improved calcium and phosphate regulation over the 24-hour dosing interval with palopegteriparatide also provided a favorable safety and tolerability profile in the phase 3 PaTHway trial [18, 19]. In addition, the effects of the sustained PTH exposure profile of palopegteriparatide on skeletal remodeling have been hypothesized to contribute to its capacity to maintain normocalcemia without calcium and active vitamin D [32].

Urine chemistries with palopegteriparatide were consistent with restoration of urinary calcium reabsorption and enhanced excretion of phosphate, which may reduce the risk for renal complications observed with conventional therapy [4, 5]. The overall control of urinary calcium excretion, maintaining levels below the upper limit of normal over 24 hours, stands in contrast to observations in patients with hypoparathyroidism treated with short-lived PTH therapies, which have not shown consistent improvements in urinary calcium. Stabilization of serum and urine calcium levels may confer potential renal benefits in this patient population [20]. In the PaTHway trial, treatment with palopegteriparatide over 52 weeks yielded a mean increase in eGFR of 9.3 mL/min/1.73 m^2^ from baseline (*P* < .0001), with 43% of participants experiencing an increase ≥ 10 mL/min/1.73 m^2^ [23]. This suggests that treatment with palopegteriparatide may not only preserve but also improve renal function in adults with chronic hypoparathyroidism. Multiple factors may drive the improvement in renal function, including sparing of conventional therapy [22, 33].

The hypocitraturia reported in a study of PTH(1-34) administered subcutaneously twice daily [34] was not observed in the PaTH Forward trial. Because citrate complexes with calcium in the renal tubule, potentially preventing stone formation, low urinary levels of citrate present an additional risk factor for renal stone formation [35].

Analysis of this sub-study is somewhat limited by its small sample size. However, given the consistency of these results with those of the larger Phase 1 study, we believe our findings provide important confirmation that the pharmacology of palopegteriparatide in people with hypoparathyroidism does not differ from that of healthy volunteers. One of the limitations of this study was the logistical challenge of standardized timing of palopegteriparatide administration among participants. Participants were enrolled across 2 study sites, and the time of day at which participants received palopegteriparatide was not standardized between sites. Additionally, the protocol did not control for external factors that may impact calcium or phosphate, including dietary intake, hydration, and activity.

In conclusion, consistent with the design features of palopegteriparatide, this sub-study of participants in the PaTH Forward trial demonstrated active PTH levels within the physiological range for 24 hours/day in adults with hypoparathyroidism treated with palopegteriparatide. The expected pharmacodynamic effects of continuous PTH exposure on specified serum and urine chemistries were confirmed and are consistent with the efficacy and safety of palopegteriparatide observed in published clinical trials [18-20].

## Data Availability

Some or all datasets generated during and/or analyzed during the current study are not publicly available but are available from the corresponding author on reasonable request.
